# Open access publishing, article downloads, and citations: randomised controlled trial

**DOI:** 10.1136/bmj.a568

**Published:** 2008-07-31

**Authors:** Philip M Davis, Bruce V Lewenstein, Daniel H Simon, James G Booth, Mathew J L Connolly

**Affiliations:** 1 Department of Communication, Cornell University, Ithaca, NY 14853, USA; 2 Department of Applied Economics and Management, Cornell University; 3 Department of Biological Statistics and Computational Biology, Cornell University; 4 Cornell University Library

## Abstract

**Objective** To measure the effect of free access to the scientific literature on article downloads and citations.

**Design** Randomised controlled trial.

**Setting** 11 journals published by the American Physiological Society.

**Participants** 1619 research articles and reviews.

**Main outcome measures** Article readership (measured as downloads of full text, PDFs, and abstracts) and number of unique visitors (internet protocol addresses). Citations to articles were gathered from the Institute for Scientific Information after one year.

**Interventions** Random assignment on online publication of articles published in 11 scientific journals to open access (treatment) or subscription access (control).

**Results** Articles assigned to open access were associated with 89% more full text downloads (95% confidence interval 76% to 103%), 42% more PDF downloads (32% to 52%), and 23% more unique visitors (16% to 30%), but 24% fewer abstract downloads (−29% to −19%) than subscription access articles in the first six months after publication. Open access articles were no more likely to be cited than subscription access articles in the first year after publication. Fifty nine per cent of open access articles (146 of 247) were cited nine to 12 months after publication compared with 63% (859 of 1372) of subscription access articles. Logistic and negative binomial regression analysis of article citation counts confirmed no citation advantage for open access articles.

**Conclusions** Open access publishing may reach more readers than subscription access publishing. No evidence was found of a citation advantage for open access articles in the first year after publication. The citation advantage from open access reported widely in the literature may be an artefact of other causes.

## Introduction

Scientists seek out publication outlets that maximise the chances of their work being cited for many reasons. Citations provide stable links to cited documents and make a public statement of intellectual recognition for the cited authors.^1^ ^2^ Citations are an indicator of the dissemination of an article in the scientific community^3^ ^4^ and provide a quantitative system for the public recognition of work by qualified peers.^5^ ^6^ Having work cited is therefore an incentive for scientists, and in many disciplines it forms the basis of a scientist’s evaluation.^6^ ^7^

In 2001 it was first reported that freely available online science proceedings garnered more than three times the average number of citations received by print articles.^8^ This “citation advantage” has since been validated in other disciplines, such as astrophysics,^9^ ^10^ ^11^ physics,^12^ mathematics,^13^ ^14^ philosophy,^13^ political science,^13^ engineering,^13^ and multidisciplinary sciences.^15^ A critical review of the literature has been published.^16^

The primary explanation offered for the citation advantage of open access articles is that freely available articles are cited more because they are read more than their subscription only counterparts. Studies of single journals have described weak but statistically significant correlations between articles downloaded from a publisher’s website and future citations,^17^ ^18^ those downloaded from a subject based repository and future citations,^19^ and those downloaded from a repository and a publisher’s website.^14^

A growing number of studies have failed to provide evidence supporting the citation advantage, leading researchers to consider alternative explanations. Some argue that open access articles are cited more because authors selectively choose articles to promote freely, or because highly cited authors disproportionately choose open access options.^14^ ^20^ ^21^ ^22^ This has been termed the self selection postulate.^20^ Self archiving an accepted manuscript in a subject based digital repository may provide additional time for these articles to be read and cited.^20^ ^21^ ^22^ In the economics literature, self archiving is much more prevalent for the most cited journals than for less cited ones.^23^ Similarly, a study of medical journals reported that the probability of an article being found on a non-publisher website was correlated with the impact factor of the journal.^24^ These findings provide evidence for the self selection postulate—that is, that prestigious articles are more likely to be made freely accessible.

Previous studies of the impact of open access on citations have used retrospective observational methods. Although this approach allows researchers to control for observable differences between open access and subscription access articles, it is unlikely to deal adequately with the self selection bias because authors may be selecting articles for open access on the basis of characteristics that are unobservable to researchers, such as expected impact or novelty of results. As a consequence, results from previous studies possibly reflect this self selection bias, which may be creating a spurious positive correlation between open access and downloads and citations. To control for self selection we carried out a randomised controlled experiment in which articles from a journal publisher’s websites were assigned to open access status or subscription access only.

## Methods

The American Physiological Society gave us permission to manipulate the access status of online articles directly from their websites. The selection and manipulation of the articles was done by the researchers without the involvement of the journals’ editors or society’s staff. From January to April 2007 we randomly assigned 247 articles published in 11 journals of the American Physiological Society to open access status (table 1). These articles formed our treatment group and were made freely available from the publisher’s website upon online publication. The control group (1372 articles) was composed of articles available to readers by subscription, which is the traditional access model for the American Physiological Society’s journals for the first year (fig 1). After the first year all articles become freely available.

**Figure fig1:**
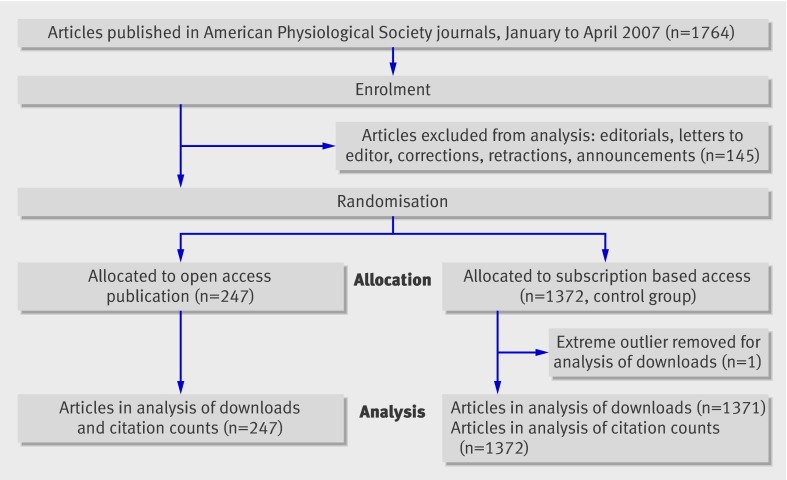
**Fig 1** Flow of study data

**Table 1 tbl1:** Description of journal dataset of American Physiological Society. Values are numbers (percentages) unless stated otherwise

Variables	No of articles	Open access articles
*American Journal of Physiology: Cell Physiology*	155	36 (23)
*American Journal of Physiology: Endocrinology and Metabolism*	147	21 (14)
*American Journal of Physiology: Gastrointestinal and Liver Physiology*	134	22 (16)
*American Journal of Physiology: Heart and Circulatory Physiology*	233	32 (14)
*American Journal of Physiology: Lung Cellular and Molecular Physiology*	109	14 (13)
*American Journal of Physiology: Regulatory, Integrative, and Comparative Physiology*	195	34 (17)
*American Journal of Physiology: Renal Physiology*	140	18 (13)
*Journal of Applied Physiology*	201	27 (13)
*Journal of Neurophysiology*	278	39 (14)
*Physiology*	11	2 (18)
*Physiological Reviews*	16	2 (13)
Total	1619	247 (15)
Categorical properties (totals):		
Research articles	1519	228 (15)
Review articles	100	19 (19)
Methods articles	29	7 (24)
Cover article	11	2 (18)
Press release	5	1 (20)
Total	1619	247 (15)
Mean (SD) continuous properties:		
Authors	5.4 (2.8)*	5.2 (2.7)†
References‡	53.4 (37.4)*	55.7 (83.4)†
Pages	9.4 (3.2)*	9.7 (6.2)†

*Open access.

†Subscription only.

‡Large standard deviation is result of few review articles, one with 1986 references.

In the randomisation we included only research articles and reviews. We excluded editorials, letters to the editor, corrections, retractions, and announcements. For those journals with sections we used a stratified random sampling technique to ensure that all categories of articles were adequately represented in the sample. Because of stratification we sampled some journals more heavily than others. To ensure an adequate sample size we experimented on four issues per journal, with the exception of *Physiology* and *Physiological Reviews*, both of which publish bimonthly and provided us with only two issues.

We tested the effect of publisher defined open access on article readership and article citations. We measured four different proxies for article readership: abstract downloads, full text (HTML) downloads, PDF downloads, and a related variable, the number of unique internet protocol addresses (an indicator of the number of unique visitors to an article). We also tested the effect of publisher defined open access on article citations; both the odds of being cited in the year after publication and the number of citations to each article.

### Sample size

The retrospective nature of previous studies did not help us to predict our expected difference in citations, although we assumed that it would be smaller than the 200-700% difference routinely reported in the literature. The American Physiological Society agreed to make 1 in 8 (15%) articles freely available. Based on a 0.7 standard deviation in log citations in the society’s journals and a 0.8 power to detect a significant difference (two sided, P=0.05), 247 open access articles allowed us to detect significant differences of about 20%. These calculations were based on equal sample sizes and a two sided test. Given that our subscription sample was much larger (n=1372) and that we did not anticipate a negative effect as a result of the open access treatment, these calculations are conservative.

### Data gathering and blinding

We were permitted to gather monthly usage statistics for each article from the publisher’s websites. HighWire Press, the online host for the American Physiological Society’s journals, maintains and periodically updates a list of known and suspected internet robots (software that crawls the internet indexing freely accessible web pages and documents) and was able to provide reports on article downloads including and excluding internet robots. Article metadata (attributes of the article) and citations were provided by the Web of Science database produced by the Institute for Scientific Information.

Free access to scientific articles can also be facilitated through self archiving, a practice in which the author (or a proxy) puts a copy of the article up on a public website, such as a personal webpage, institutional repository, or subject based repository. To get an estimate of the effect of self archiving, we wrote a Perl script to search for freely available PDF copies of articles anywhere on the internet (ignoring the publisher’s website). Our search algorithm was designed to identify as many instances of self archiving as possible while minimising the number of false positives. 

Before randomisation we emailed corresponding authors to notify them of the trial and to provide them with an opportunity to opt out. No one opted out. An open green lock on the publisher’s table of contents page indicated articles made freely available.

### Statistical analysis

We used linear regression to estimate the effect of open access on article downloads and unique visitors. Our outcome measures were downloads of abstracts, full text (HTML), and PDFs, and the number of unique internet protocol addresses (a proxy for the number of unique visitors). Because of known skewness in these outcomes,^25^ we log transformed these variables. Our principal explanatory variable was the open access treatment (a dummy variable). We controlled for three important indicators of quality that could influence downloads: whether the article was self archived, featured on the front cover of the journal, and received a press release from the journal or society. We also controlled for several other attributes that could influence downloads: article type (review, methods), number of authors, whether any of the authors were based in the United States, the number of references, the length of the article (in pages), and the journal impact factor. As articles are published within issues we nested the issue variable within the journal variable. Journals are considered a random variable and, by necessity, so is the issue variable. We are not concerned with estimating the effect of each of the 11 journals participating in this trial but consider journals to explain some variance in the model.

On 2 January 2008 we retrieved the number of article citations from the Web of Science. As our trial included articles published at different times (January to April 2007), we dealt with the disparity in age of articles by using a numerical indicator for each issue of a journal.

We estimated the effect of open access on citation counts using a negative binomial regression model with the same set of explanatory variables previously described. We chose the negative binomial regression model over a linear regression model because our citation dataset included a lot of zeros, and because the negative binomial regression model resulted in a better fit to the data than a linear regression model. The negative binomial regression model is appropriate for count data and is similar to the Poisson regression model except that it can work with over-dispersion in the data.^26^

Finally, we used a logistic regression model to estimate the effect of open access on the odds of being cited, with the same set of explanatory variables employed in the download and negative binomial regression citation model. We used SAS software JMP version 7 for the linear regression and logistic regression and Stata version 10 for the negative binomial regression. Two sided significance tests were used throughout.

## Results

Figure 2 shows the effect of open access on article downloads and unique visitors in the six months after publication. Full text downloads were 89% higher (95% confidence interval 76% to 103%, P<0.001), PDF downloads 42% higher (32% to 52%, P<0.001), and unique visitors 23% higher (16% to 30%, P<0.001) for open access articles than for subscription access articles. Abstract downloads were 24% lower (−29% to −19%, P<0.001) for open access articles. Moreover, the effect of open access on article downloads seems to be increasing with time (see supplementary figure at http://hdl.handle.net/1813/11049).

**Figure fig2:**
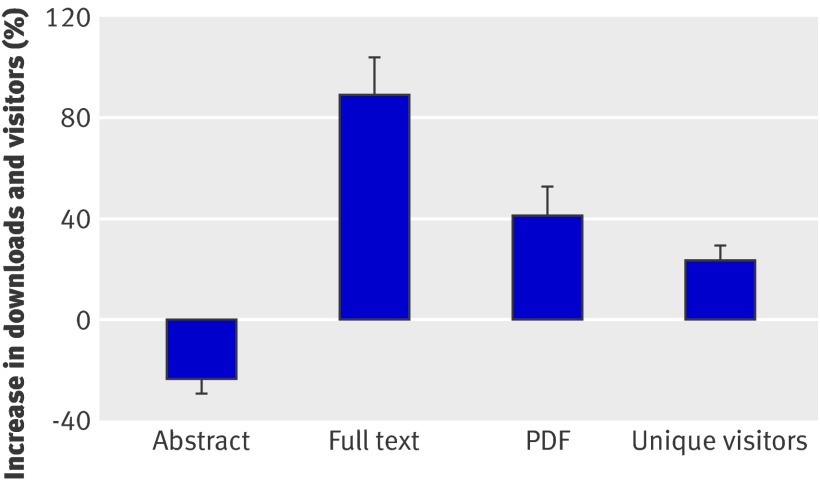
**Fig 2** Percentage differences (95% confidence intervals) in downloads of open access articles (n=247) and subscription access articles (n=1371) during the first six months after publication. Downloads from known internet robots are excluded

For open access articles, known internet robots could account for an additional 83% full text downloads, 5% additional PDF downloads, 4% additional unique visitors, and a 12% reduction of all abstract downloads. 

Regression analysis showed that several characteristics of articles had as much, or more, of an effect on article downloads as free access (table 2). For example, being a review article had the largest effect on PDF downloads (100% increase, 95% confidence interval 74% to 131%). Having an article featured in a press release from the publisher increased PDF downloads by 65% (7% to 156%), and having an article featured on the front cover of the journal increased PDF downloads by 64% (21% to 121%). Longer articles, articles with more references, and those published in journals with higher impact factors had significantly more downloads.

**Table 2 tbl2:** Linear regression output reporting independent variable effects on PDF downloads for six months after publication

Variables	% Regression coefficient (95% CI)	P value
Fixed effect coefficient:		
Open access	43 (34 to 53)	<0.001
Cover article	64 (21 to 121)	0.001
Press release	65 (7 to 156)	0.024
Self archived	6 (−6 to 19)	0.361
Review article	100 (74 to 131)	<0.001
Methods article	6 (−12 to 28%)	0.509
Any author from USA	0 (−5 to 5)	0.950
No of authors*	5 (0 to 10)	0.069
No of references*	23 (13 to 35)	<0.001
Article length (pages)*	17 (5 to 31)	0.005
Journal impact factor*	59 (28 to 97)	0.000
Intercept	1627 (1035 to 2529)	<0.001
Random effects coefficient:		
Journal	2 (0 to 5)†	9‡
Issue	0 (0 to 1)†	1‡
Residual	28 (26 to 30)†	90‡
Total	31‡	100‡

F_31, 1350_=37.5, P<0.001, R^2^=0.42.

*Log transformed.

†Variance component (%) (95% confidence interval).

‡Percentage of variance explained.

Twenty instances of self archiving could be identified, of which 18 were final copies from the publisher and two were authors’ final manuscripts. The estimated effect of self archiving was positive on PDF downloads, although non-significant (6%, −6% to 19%; P=0.36), and essentially zero for full text downloads (−1%, −23% to 27%; P=0.95).

Of the 247 articles randomly assigned to open access status, 59% (n=146) were cited after 9-12 months compared with 63% (859 of 1372) of subscription access articles.

The negative binomial regression model estimated that open access reduced expected citation counts by 5% (incident rate ratio 0.95, 95% confidence interval 0.81 to 1.10; P=0.484) and that self archiving reduced expected citation counts by about 10% (0.90, 0.53 to 1.55; P=0.716), although neither of these estimates are significantly different from zero (table 3).

**Table 3 tbl3:** Negative binomial regression output reporting independent variable effects on citations to articles aged 9 to 12 months

Coefficient	Incidence rate ratio (95% CI)	P value
Open access	0.95 (0.81 to 1.10)	0.484
Cover article	0.93 (0.52 to 1.64)	0.789
Press release	1.23 (0.50 to 3.05)	0.654
Self archived	0.90 (0.53 to 1.55)	0.716
Review article	1.34 (1.01 to 1.78)	0.041
Methods article	0.62 (0.37 to 1.06)	0.079
Any author from USA	1.17 (1.04 to 1.32)	0.007
No of authors*	1.13 (1.01 to 1.26)	0.030
No of references*	1.42 (1.17 to 1.72)	<0.001
Article length (pages)*	1.09 (0.86 to 1.38)	0.483
Journal impact factor*	1.39 (1.10 to 1.76)	0.006
Issue	1.26 (1.20 to 1.33)	<0.001

χ^2^_12, 1607_=313.14, P<0.001, pseudo R^2^=0.06, α for dispersion 0.43 (95% confidence interval 0.35 to 0.54).

*Log transformed.

A supplementary logistic regression analysis based on the same set of variables for articles estimated that open access publishing reduced the expected odds of being cited by about 13% (odds ratio 0.87, 95% confidence interval 0.66 to 1.17; P=0.36, see supplementary table at http://hdl.handle.net/1813/11049), although this effect was not statistically significant.

## Discussion

Strong evidence suggests that open access increases the readership of articles but has no effect on the number of citations in the first year after publication. These findings were based on a randomised controlled trial of 11 journals published by the American Physiological Society.

Although we undoubtedly missed a substantial amount of citation activity that occurred after these initial months, we believe that our time frame was sufficient to detect a citation advantage, if one exists. A study of author sponsored open access in the *Proceedings of the National Academies of Sciences* reported large, significant differences in only four to 10 months after publication.^15^ Future analysis will test whether our conclusions hold over a longer observation period.

Previous studies have relied on retrospective and uncontrolled methods to study the effects of open access. As a result they may have confused causes and effects (open access may be the result of more citable papers being made freely available) or have been unable to control for the effect of multiple unmeasured variables. A randomised controlled design enabled us to measure more accurately the effect of open access on readership and citations independently of other confounding effects.

Our finding that open access does not result in more article citations challenges established dogma^8^ ^9^ ^10^ ^11^ ^12^ ^13^ ^15^ and suggests that the citation advantage associated with open access may be an artefact of other explanations such as self selection.

Whereas we expect a general positive association between readership and citations,^14^ ^17^ ^18^ ^19^ we believe that our results are consistent with the stratification of readers of scientific journals. To contribute meaningfully to the scientific literature, access to resources (equipment, trained people, and money) as well as to the relevant literature is normally required. These two requirements are highly associated and concentrated among the elite research institutions around the world.^7^ ^27^ That we observed an increase in readership and visitors to open access articles but no citation advantage suggests that the increase in readership is taking place outside the community of core authors.

Although we need to be careful not to equate article downloads with readership (we have no idea whether downloaded articles are actually read), measuring success by only counting citations may miss the broader impact of the free dissemination of scientific results.

The increase in full text downloads for open access articles in the first six months after publication (fig 2) suggests that the primary benefit to the non-subscriber community is in browsing, as opposed to printing or saving, which would have been indicated by a commensurate increase in PDF downloads. The fact that internet robots were responsible for so much of the initial increase in full text downloads (an additional 83%) compared with PDF downloads (an additional 5%) implies that internet search engines are helping to direct non-subscribers to free journal content. Lastly, the reduction in abstract downloads for open access articles suggests that non-subscribers were probably substituting free full text or PDF downloads (when available) for abstract downloads.

We studied the effect of providing free access to scientific literature directly from the publisher’s website; however, scientific information can be disseminated in many ways. Although the author-pays open access model has received the greatest amount of attention, we should not ignore the many creative access models that publishers use: the delayed access model, with all articles becoming freely available after a defined period after publication; the selective access model, with certain types of articles (for example, original research) being made freely available in subscription access journals; or variations of the models. Most scientific publishers allow authors to post manuscripts of their articles on their own website or in their institution’s digital repository. Funding agencies, such as the National Institutes of Health (United States) and the Wellcome Trust (United Kingdom) have policies for self archiving. One model for publication may not fit the needs of all stakeholders.^28^

### Unanswered questions and future research

The discussion over access and its effects on citation behaviour assumes that articles are read before they are cited. Studies on the propagation of citation errors suggest that many citations are merely copied from the papers of other articles.^29^ ^30^ ^31^ Given the common behaviour of citing from the abstract (normally available free), the act of citation does not necessarily depend on access to the article. Secondly, the rhetorical dichotomy of “open” access compared with “closed” access does not recognise the degree of sharing that takes place among an informal network of authors, libraries, and readers. Subscription barriers are, in reality, porous.

Our citation counts are limited to those journals indexed by Web of Science. Because this database focuses on covering the core journals in a particular discipline, we missed citations in articles published in peripheral journals.

We measured the number of unique internet protocol addresses as a proxy for the number of visitors to an article. We implied that the difference in number of visitors between open access and subscription based articles (in our case 23%) represents the size of the non-subscriber population. A more direct (although more laborious) method of calculating access by non-subscribers would be to analyse the log transaction files of the publisher and to compare the list of internet protocol addresses from subscribing institutions with the total list of internet protocol addresses. Because of confidentiality issues we did not have access to the raw transaction logs.

Open access articles on the American Physiological Society’s journals website are indicated by an open green lock on the table of contents page. Although icons representing access status are a common feature of most journals’ websites, these may signal something about the quality of the article to potential readers, especially as open access articles have been associated with a large citation advantage.^8^ ^9^ ^10^ ^11^ ^12^ ^13^ ^14^ ^15^ As a result, readers may have developed a heuristic that associates open access articles with higher quality. This quality signal could have been imparted to those randomly assigned to open access articles in our study and created a positive bias on download counts.

Conversely, we were told by the publisher that most readers never view the table of contents pages. Most people are referred directly to the article by search engines, such as Google, or through the linked references of other articles. Subject indexes, such as PubMed, did not provide an indication of which articles were randomly selected for open access. It is likely that few readers were aware that they were viewing a free article.

Finally, we do not understand whether providing open access to articles had any effect on the behaviour of the authors as they promoted their work to the wider community. We are currently carrying out similar randomised experiments with other journals in an environment where neither authors nor readers are aware of the access status of the article.

Research suggests that a publisher’s web interface can influence the accessibility and use of online articles^32^ ^33^; hence we are studying journals published on a single online platform (HighWire Press). We have recently expanded our open access experiment to include an additional 25 journals hosted by HighWire in the disciplines of multidisciplinary sciences, biology, medicine, social sciences, and the humanities. This will allow us to assess whether our results generalise to a broader set of disciplines. We are also observing the performance of 10 control journals that allow author sponsored open access publishing. This will help us to explore which confounding variables may explain the citation advantage that has been widely reported in the literature.

**What is already known on this topic**

Studies suggest that open access articles are cited more often than subscription access ones
These claims have not been validated in a randomised controlled trial

**What this study adds**

Open access articles had more downloads but exhibited no increase in citations in the year after publication
Open access publishing may reach more readers than subscription access publishing
The citation advantage of open access may be an artefact of other causes

## Notes

We thank Bill Arms, Paul Ginsparg, Simeon Warner, and Suzanne Cohen at Cornell University for their critical feedback on our experiment.

Ethical approval: This study was approved by the institutional review board at Cornell University.

Provenance and peer review: Not commissioned; externally peer reviewed.

## Notes

**Cite this as:** *BMJ* 2008;337:a568
